# Supplementary material for: Comparative Analysis of Platelet-Derived Extracellular Vesicles Using Flow Cytometry and Nanoparticle Tracking Analysis
Source: Int J Mol Sci. 2021 Apr 7;22(8):3839. doi: 10.3390/ijms22083839 (PMC8068037; doi:10.3390/ijms22083839)
Supplement: Supplementary file 1 [file ijms-22-03839-s001.pdf]

Supplementary Material

Comparative Analysis of Platelet-Derived Extracellular Vesicles Using Flow Cytometry and Nanoparticle Tracking Analysis

Sobha Karuthedom George[1], Lucia Lauková[1], René Weiss[1], Vladislav Semak[1], Birgit Fendl[1], Victor U. Weiss[2], Stephanie Steinberger[2], Günter Allmaier[2], Carla Tripisciano[1] and Viktoria Weber[1*]

[1]Center for Biomedical Technology, Department for Biomedical Research, Danube University Krems, Austria
[2]Institute of Chemical Technologies and Analytics, TU Wien, Vienna, Austria

*Correspondence: viktoria.weber@donau-uni.ac.at; Tel.: +43 2732 893 2632

Supplementary Table S1. Fluorochrome conjugates and dyes used for flow cytometry and nanoparticle tracking analysis in fluorescence mode. n.a., not applicable.

Flow Cytometry				
	**Marker for**	**Fluorochrome**	**Abbreviation**	**Supplier**
**anti-CD41 monoclonal antibody**	platelets	Phycoerythrin Cyanin 7	PC7	Beckman Coulter
**lactadherin**	phosphatidylserine	Fluorescein Isothiocyanate	FITC	Haematologic Technologies
**Nanoparticle Tracking Analysis**				
	**Marker for**	**Fluorochrome**	**Abbreviation**	**Supplier**
**CellMask™ Orange**	lipid membrane	n.a.	n.a.	Invitrogen
**lactadherin**	phosphatidylserine	Alexa Fluor® 555	AF555	Haematologic Technologies

Supplementary Figure S1

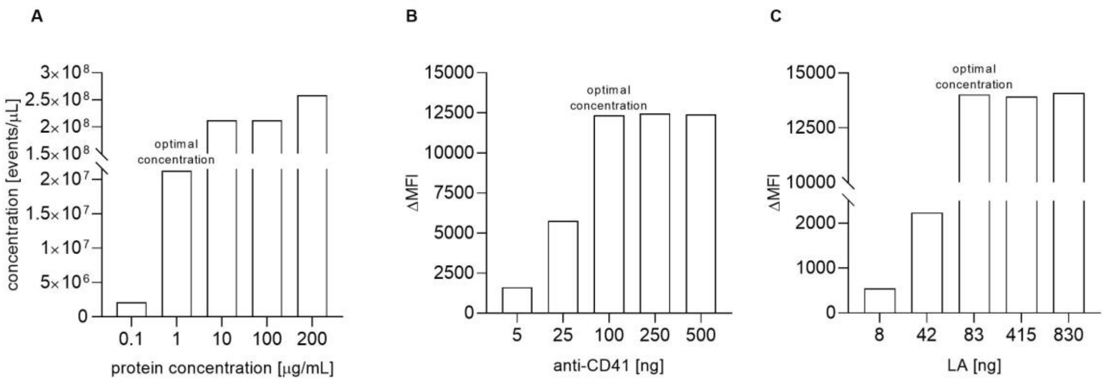

Supplementary Figure S1. Optimization of the staining protocol for flow cytometry. (A) Determination of optimal protein concentration. Different dilutions of isolated platelet-derived EVs containing 0.1, 1, 10, 100 and 200 µg protein/mL were tested. Aliquots of 100µl of diluted EV suspensions were characterized using the CytoFLEX LX flow cytometer (violet side scatter *vs*. forward scatter), and the EV gate was set as described in the Methods section and shown in Fig.1 of the main manuscript. A dilution of 1µg EV protein/mL was identified as optimal for further experiments, as higher concentrations showed swarming or coincident event detection. **(B)** Determination of optimal antibody concentration for CD41 staining. Different amounts of PC7-labeled anti-CD41 antibody were tested: 5, 25, 100, 250 and 500 ng. Stained was performed as described in the Methods section of the main manuscript. Staining of 1 µg/mL EV concentration with 100 ng anti-CD41-PC7 was chosen due to its optimal saturating concentration. **(C)** Determination of the optimal antibody concentration for lactadherin (LA) staining. Different amounts of LA were tested: 8, 42, 83, 415 and 830 ng. Staining was performed as described in the Methods section of the main manuscript. Staining of 1 µg/mL EV concentration with 83 ng lactadherin was chosen as optimal (saturating) antibody concentration. Results are depicted as delta median fluorescence intensity (ΔMFI), i.e. difference between the specific antibody staining and the respective fluorochrome-labeled reagent control. Flow speed: 10 µL/min.

Supplementary Figure S2

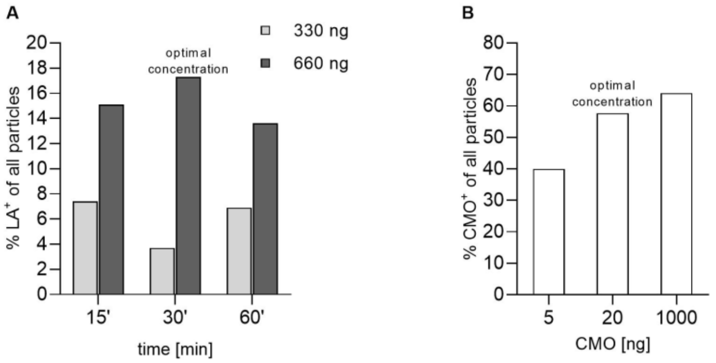

Supplementary Figure S2. Optimization of the staining protocol for fluorescence-based nanoparticle tracking analysis. Staining protocols were optimized by testing different EV-to-dye ratios for **(A)** For the staining of EVs exposing phosphatidylserine, 330 ng and 660 ng LA-AF555 were used to label 10 µg sample protein. Additionally, different staining times (15 min, 30 min, and 60 min) were compared. Staining for 30 min with 660 ng LA-AF555 was chosen for all further experiments due to minimal background signal with optimal staining efficiency. **(B)** For CellMask Orange staining, dye concentrations of 5, 20, and 1000 ng were used to label 10 µg sample protein. A comparison of 30 *vs*. 60 min staining time did not yield a difference in CMO staining efficiency. Thus, a staining time of 30 min was chosen for all further experiments, along with 20 ng CMO per 10 µg sample protein, which yielded optimal staining results with minimal background noise.

Text S1. Fourier-transform infrared spectroscopy of platelet-derived EVs

EV samples (5 μL) were mounted on the ZnSe ATR crystal and thin dry films were obtained by slow evaporation of the solvent under ambient conditions (drop-coating deposition). For recording of full FTIR spectra (650 to 4000 cm^{-1}), 64 scans were collected at a nominal resolution of 4 cm^{-1}. The spectral evaluation protocol[1] consists of the following successive steps:

1) Background subtraction.
2) All types of software corrections (e.g. baseline, ATR) and spectra smoothing procedures were omitted and raw spectra were used for peak quantifications.
3) Determination of protein content by measuring the corrected area under the curve (AUC) of the amide I band region (1600-1700 cm^{-1}).
4) Determination of the lipid content by measuring the corrected area under the curve (AUC) of the aliphatic C-H stretching vibration bands region (2700 to 3000 cm^{-1}).
5) Calculation of spectroscopic protein-to-lipid (P/L) ratio according to the equation:

$$P/L = \frac{AUC\ (1600-1700\ cm^{-1})}{AUC\ (2700-3000\ cm^{-1})}.$$

The curve fitting procedure (instead of integration of deconvoluted peak) was employed to determine the AUC of the amide I band (1600-1700 cm^{-1}). This method is preferred, as in case of EVs samples isolated by centrifugation, potentially co-isolated contaminants (e.g. lipoproteins, protein complexes) exhibit bands around 1600 cm^{-1}. The area was restricted to 1700 cm^{-1} in order not to cover the carbonyl stretching peak (≈1740 cm^{-1}) of lipid-related ester bonds. The lipid content was determined analogically by measuring the corrected AUC of the C-H stretching bands in the 2700-3000 cm^{-1} region.

[1] Inspired by Mihály, J.; Deák, R.; Szigyártó, I.C.; Bóta, A.; Beke-Somfai, T.; Varga, Z. Characterization of extracellular vesicles by IR spectroscopy: Fast and simple classification based on amide and CH stretching vibrations. *Biochim. Biophys. Acta - Biomembr.* 2017, *1859*, 459–466, doi:10.1016/j.bbamem.2016.12.005.

Supplementary Figure S3

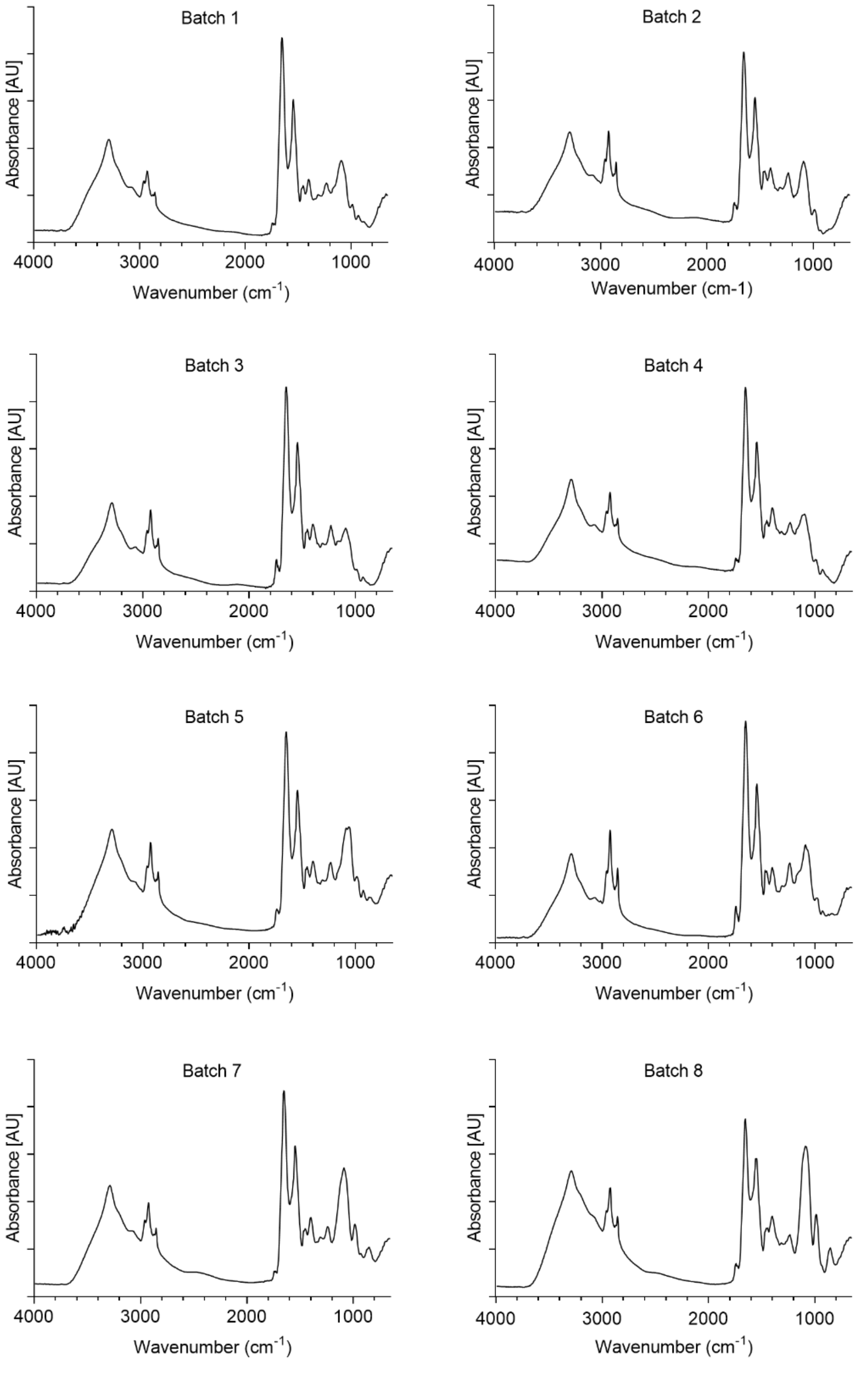

Supplementary Figure S3. Characterization of platelet-derived EVs by Fourier-transform infrared spectroscopy (FT-IR). ATR/FT-IR spectra of 8 batches of EVs enriched from platelet concentrate. AU, arbitrary units.

Supplementary Table S2. Frequencies (cm^{-1}) of the most important FT-IR absorption bands of platelet-derived EV. [2,3,4,5]

Range	Assignment	Comments
**≈3287**	N-H st<br>O-H st	Strong brad peak. Overlapping of N-H stretching vibrations of the peptide groups (amide A) and O-H stretching vibrational bands.
**≈2923**<br>**≈2852**	$-CH_2-$ st as<br>$-CH_2-$ st sy	Aliphatic acyl $-CH_2-$ groups of lipids.
**≈1740**	C=O st	Aliphatic ester group of triglycerides, phospholipids and/or cholesterol esters.
**≈1652**	C=O st	Key amide absorption band (amide I) of NHC=O stretching vibrations of protein peptide backbone.
**≈1546**	N-C=O st sy<br>N-H δ	Second absorption band of amides (amide II) of superposed N-H bending vibrations and N-C=O symmetric stretching bands of peptide NHC=O groups.
**1400-1500**	CH_3 δ<br>CH_2 δ	Bands belonging to bending vibrations of aliphatic $-CH_2-$ and $-CH_3$ groups.
**1300-800**	P=O st<br>P-OH δ	All peaks originating from EVs in this region are overlapped by strong broad phosphate absorption bands of the PBS.

Abbreviations: st stretching, as asymmetric, sy symmetric, δ bending.

[2] Mihály, J.; Deák, R.; Szigyártó, I.C.; Bóta, A.; Beke-Somfai, T.; Varga, Z. Characterization of extracellular vesicles by IR spectroscopy: Fast and simple classification based on amide and CH stretching vibrations. *Biochim. Biophys. Acta - Biomembr.* 2017, *1859*, 459–466, doi:10.1016/j.bbamem.2016.12.005.

[3] Szentirmai, V.; Wacha, A.; Németh, C.; Kitka, D.; Rácz, A.; Héberger, K.; Mihály, J.; Varga, Z. Reagent-free total protein quantification of intact extracellular vesicles by attenuated total reflection Fourier transform infrared (ATR-FTIR) spectroscopy. *Anal. Bioanal. Chem.* 2020, *412*, 4619–4628, doi:10.1007/s00216-020-02711-8.

[4] Paolini, L.; Federici, S.; Consoli, G.; Arceri, D.; Radeghieri, A.; Alessandri, I.; Bergese, P. Fourier-transform Infrared (FT-IR) spectroscopy fingerprints subpopulations of extracellular vesicles of different sizes and cellular origin. *J. Extracell. Vesicles* 2020, *9*, doi:10.1080/20013078.2020.1741174.

5 Kitka, D.; Mihály, J.; Fraikin, J.L.; Beke-Somfai, T.; Varga, Z. Detection and phenotyping of extracellular vesicles by size exclusion chromatography coupled with on-line fluorescence detection. *Sci. Rep.* 2019, 9, 19868, doi:10.1038/s41598-019-56375-1.
